# Enhancing forensic clinical competence through scenario-based simulation: A comparative study of educational outcomes in Chinese medical students

**DOI:** 10.1371/journal.pone.0336273

**Published:** 2025-11-13

**Authors:** Jiaqi Li, Chunmei Hou, Dan Yang, Xue Peng, Yingli Song, Bo Cao, Haixia Li

**Affiliations:** 1 Department of Forensic Medicine, School of Basic Medical Sciences, Harbin Medical University, Harbin, Heilongjiang, China; 2 Functional Experimental Teaching Centre of Harbin Medical University, Harbin, Heilongjiang, China; 3 Department of Histology and Embryology, School of Basic Medical Sciences, Harbin Medical University, Harbin, Heilongjiang, China; Florida International University, UNITED STATES OF AMERICA

## Abstract

**Introduction:**

Forensic clinical medicine examines injuries in living individuals and plays a critical role in criminal violence cases, trauma compensation, and judicial rulings. In China, this field contributes substantially to upholding social justice. Current educational approaches struggle to bridge the gap between theory and practice, largely due to privacy constraints during forensic examinations, leaving students ill-prepared to assess real cases and identify key evaluation criteria. Scenario-based simulation training presents a promising alternative to conventional case-based teaching.

**Methods:**

We compared the efficacy of traditional case-based teaching with scenario-based simulation in a forensic clinical medicine course. Two cohorts of undergraduate forensic science students from consecutive academic years underwent each teaching method. Post-course assessments included theoretical examinations and practical evaluations as quantitative measures of knowledge acquisition. Professional instructors graded students’ performance, while self-evaluation surveys captured learning experiences.

**Results:**

Scenario-based simulation teaching yielded superior learning outcomes. The simulation group (2020 cohort) achieved higher scores than the traditional group (2019 cohort) in both theoretical (*p < 0.05) and practical assessments, with notably better knowledge retention (subjective questions: **p < 0.01; objective questions: *p < 0.05). The simulation group also demonstrated stronger theory-practice integration (*r* = 0.9622 *vs.* 0.9115). Instructor evaluations (n = 11) demonstrated that scenario-based simulation teaching improved students’ learning motivation (81.8%), theoretical application (72.7%), communication skills (81.8%), analytical abilities (63.6%), and teamwork competencies (54.5%). All instructors reported enhanced professional image, with 90.9% noting increased teaching motivation. Student self-assessments reinforced these results: over 80% of the simulation group reported gains in theoretical learning effectiveness, analytical/operational capabilities, and professional self-assurance, versus ≤31.9% in the traditional group. Classroom metrics favored the simulation group (self-study interest: 65% *vs.* 45.5%; engagement: 90% *vs.* 45.5%; collaboration: 80% *vs.* 36.4%), though 10% cited challenges with preparatory workload.

**Conclusion:**

Scenario-based simulation teaching significantly enhances forensic clinical education by strengthening the connection between theory and practice while improving student competencies. Addressing teaching costs and adapting to student needs will further refine its effectiveness.

## Introduction

Forensic Clinical Medicine, or Clinical Forensic Medicine, examines criminal violence and associated trauma, serving a vital role in victim injury assessment and perpetrator behavior analysis. Its significance spans individual rights protection to broader societal stability and judicial integrity [1–3]. Yet forensic clinical education confronts substantial obstacles [4]. Data from multiple countries reveal that recent graduates often struggle to conduct independent forensic evaluations, primarily due to mismatches between academic training and professional demands [5], inadequate exposure to real-case complexities [6] and the inability of conventional methods to adapt to evolving forensic standards [7]. In China, while legal reforms have raised expectations for forensic evaluation quality, traditional pedagogy fails to replicate authentic work conditions. Restricted access to real case materials further limits students’ practical training, leaving them underprepared in communication, diagnostic reasoning, and case management.

To mitigate these gaps, researchers propose scenario-based simulation as a pivotal educational intervention [8]. Simulation-based medical education (SBME), increasingly adopted in medical training, employs simulated tools and environments to replicate clinical settings. This approach enables risk-free experiential learning while maintaining fidelity to real-world challenges, allowing trainees to confront practice scenarios without actual consequences.

Empirical evidence confirms that scenario-based simulation elevates clinical performance. Yan et al. demonstrated that role-playing scenarios in physiology courses enhance medical students’ active learning and knowledge application, reducing theory-practice disparities [7]. Kaddoura et al. validated its efficacy in bridging theoretical and clinical gaps in nursing education [9], and surgical residents trained on simulators outperform peers in operative settings [10]. The method also improves procedural mastery, teamwork, and communication [11]. Notably, simulation stimulates autonomous learning and collaborative skills: group interactions deepen engagement, while reflective practice consolidates knowledge through iterative cycles of application and discussion [11].

Given these demonstrated benefits, scenario-based simulation holds particular promise for forensic clinical education. It circumvents privacy constraints via simulated cases, permits risk-free repetition, and integrates cross-disciplinary competencies. This study evaluates its implementation in undergraduate forensic courses by measuring knowledge gains through examinations, assessing skill development via instructor evaluations, and analyzing longitudinal outcomes through student feedback.

## Methods

### 1. Study participants

This study included forensic medicine students from Harbin Medical University’s 2019 (n = 22) and 2020 (n = 20) cohorts. All participants had completed uniform pre-admission education, passed the national university entrance examination, and undergone standardized clinical medicine training and internships before starting forensic medicine coursework. They began the Forensic Clinical Medicine course in their eighth semester. Data collection was conducted from July 18 to July 20, 2025. This study was approved by the Ethics Committee of Harbin Medical University. All participants voluntarily completed an anonymous questionnaire. Data collection strictly adhered to privacy protection principles, with no personal identification information involved.

### 2. Study protocol

**Fig 1:**
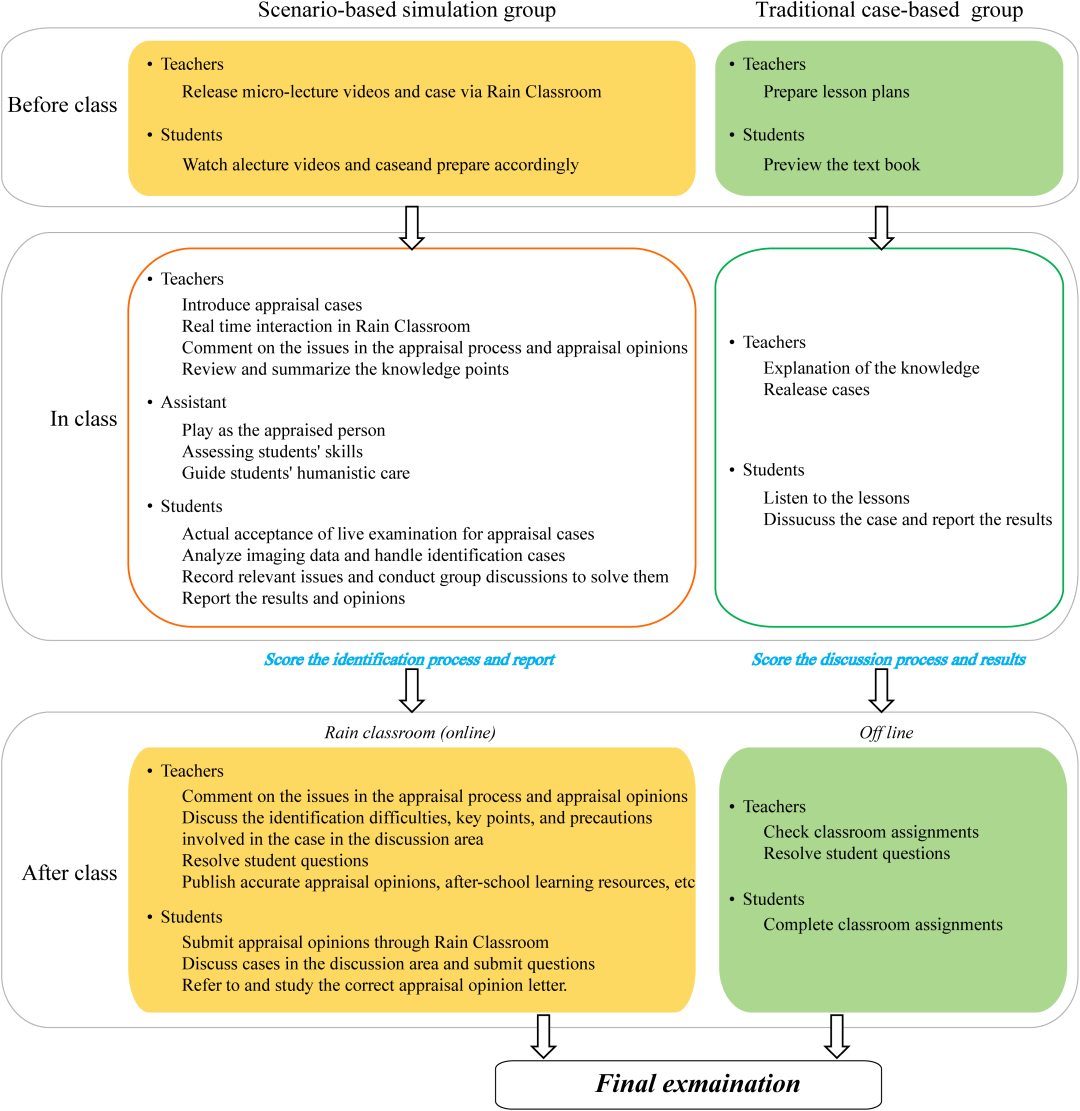
Research flow.

The 2019 cohort received traditional case-based teaching (tradition group), while the 2020 cohort participated in scenario-based simulation teaching (simulation group). Both groups followed the same syllabus, used identical teaching cases, and were instructed by the same faculty. Fig 1 outlines the study protocol.

#### 1) Scenario-based simulation teaching group.

The Forensic Clinical Medicine course employed exclusively scenario-based simulations. Rain Classroom, an online learning platform, facilitated virtual classroom activities. Students were divided into four groups of five members each.

**a) Pre-class preparation:**

Students received standardized self-directed learning materials, including:

i Instructor-curated cases adapted from course content and real-world examples
ii Faculty-recorded instructional videos on forensic clinical evaluation
iii Required independent study of foundational knowledge and operational procedures before class

**b) In-class activities:**

i Faculty presented complex case scenarios, with teaching assistants role-playing as evaluation subjects
ii Student groups analyzed and discussed cases using newly acquired knowledge
iii Teaching assistants provided observational guidance
iv Faculty evaluated and scored student performance

#### 2) Traditional case-based teaching group:.

This group received hybrid instruction combining lectures with case-based teaching. Faculty first delivered systematic theoretical instruction, then introduced representative cases to connect concepts with practical applications. During case discussions, students worked in four groups of five to six members each.

#### 3. Teaching effectiveness evaluation.

1) **Assessment Methods**

Post-course evaluations employed multiple approaches:

  a) Theoretical Examination
    Assessed mastery of forensic clinical theory through:

i Multiple-choice questions (objective items)
ii Short-answer questions
iii Case analysis (subjective items)
iv Weighting at 60% of final examination score

  b) Forensic Clinical Evaluation Skills Assessment
    Student teams demonstrated:

i Case analysis (protocol development, file review)
ii Practical evaluation (report completion, issue identification), assessing operational skills, decision-making, and teamwork
iii Weighted at 40% of the final examination score

  c) Questionnaire Survey
    Anonymous questionnaires evaluated:

i Student satisfaction with teaching methods
ii Self-assessed learning outcomes

2) **Evaluation metrics**

Teaching effectiveness comparisons included:

a) Analysis of theoretical and practical examination scores between groups
b) Expert instructor assessments of operational competencies
c) Correlation analysis between regular academic performance (participation, assignments) and final examination results
d) Anonymous student feedback from both teaching groups

### 4. Statistical analysis

Examination scores are presented as mean ± SEM. GraphPad Prism 8.0 (GraphPad Software, San Diego, CA) performed Student’s *t*-tests for intergroup comparisons and Pearson correlation analysis between academic performance and examination scores. Statistical significance was set at *p* < 0.05 (two-tailed).

## Results

### 1. Scenario-based teaching enhances knowledge and skill acquisition

**Fig 2:**
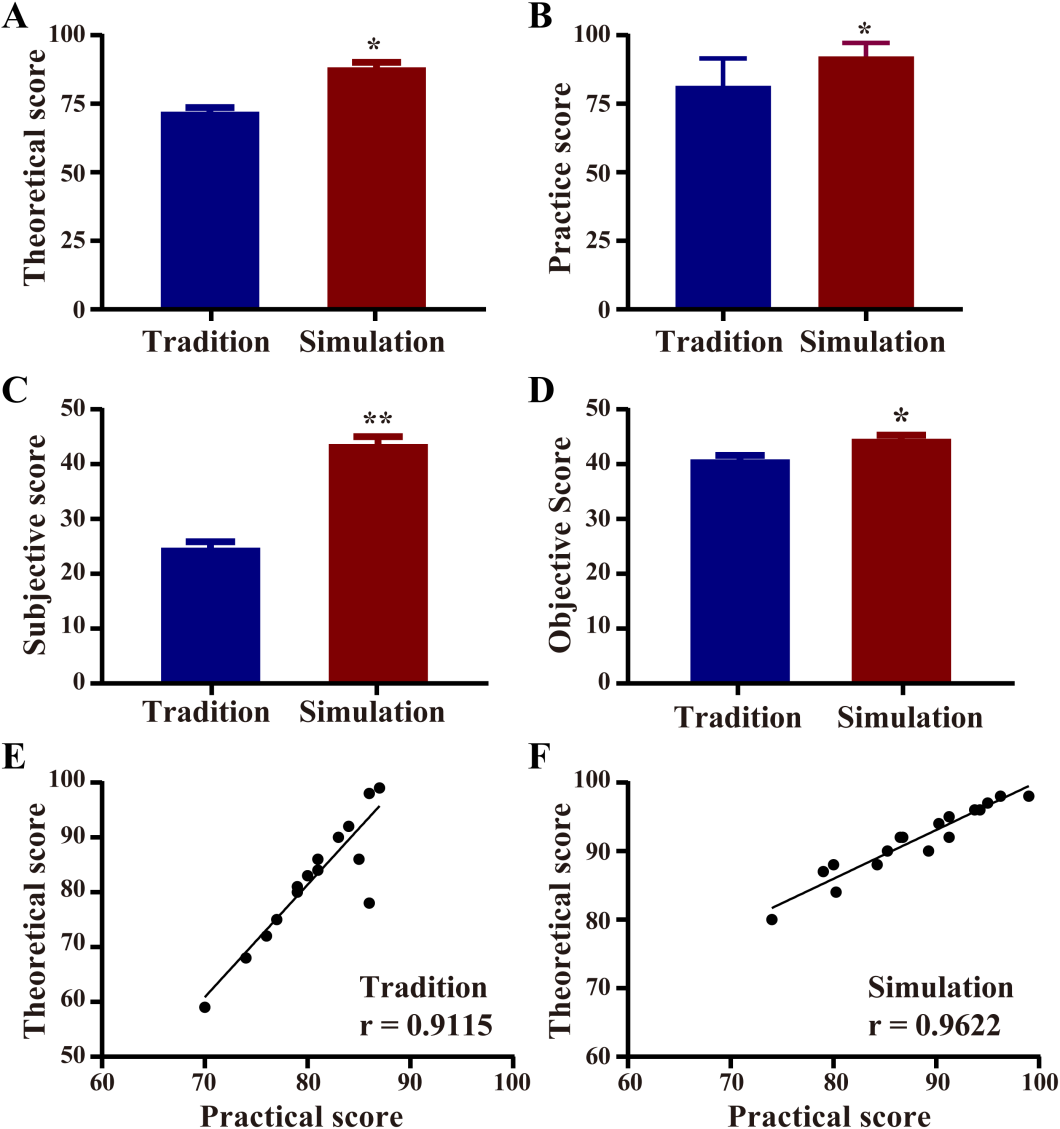
Theoretical and practical exam performance and their correlation. (A) Theoretical exam scores. (B) Practical assessment scores. (C) Subjective and (D) objective components of the theoretical exam. Correlations between theoretical and practical performance for the 2019 (E, n = 22) and 2020 (F, n = 20) cohorts. Data represent mean ± SEM; Student’s t-test compared results with the traditional group (*p < 0.05, **p < 0.01).

Theoretical and practical examination scores were used to evaluate learning outcomes. Final examination performance comparisons showed that students in the simulation group (2020 cohort) scored significantly higher in both theoretical and practical assessments than those in the traditional group (2019 cohort) (Figs 2A and 2B; *p < 0.05; Figs 2A: Mean difference ± SEM = 16.23 ± 2.377; Figs 2B: Mean difference ± SEM = 10.70 ± 2.478), confirming the superior efficacy of scenario-based simulation in forensic clinical education.

To assess knowledge retention, comprehension, and application, theoretical examination items were divided into objective (recall and foundational knowledge) and subjective (analytical and applied skills) components. The simulation group outperformed the traditional group in both subjective (Fig 2C, **p < 0.01, Mean difference ± SEM = 18.90 ± 1.713) and objective questions (Fig 2D, *p < 0.05, Mean difference ± SEM = 3.773 ± 1.015), indicating improved mastery of foundational concepts and higher-order cognitive skills.

Pearson correlation analysis revealed a stronger association between theoretical and practical scores in the simulation group (r = 0.9622, 95% CI = 0.9051–0.9852) than in the traditional group (r = 0.9115, 95% CI = 0.7958–0.9630) (Figs 2E and 2F). This suggests that scenario-based teaching fosters better integration of theory and practice, enabling students to apply conceptual knowledge more effectively in clinical contexts.

Unlike the tradition group, which exhibited uniform performance, the simulation group displayed polarized academic outcomes. Follow-up interviews indicated that engaged students benefited academically, with higher scores and multidimensional skill development, while resistant students underperformed. Despite this variation, the simulation cohort achieved significantly better overall results (Table 1).

**Table 1:** The average scores of students in the traditional group and Simulation group.

Project	Tradition	Simulation
**Average practical score**	83.14	92.25
**Average exam score**	72.09	88.83
**Median practical score**	81.50	94.00
**Median exam score**	71.00	90.25
**Highest exam score** ^**a**^	83.00/82.00/82.00	99.00/96.25/95.00
**Minimum exam score** ^**b**^	56.00/62.00/62.00	74.00/79.00/80.00
**Highest practical score** ^**a**^	99.40/97.75/97.00	98.00/98.00/97.00
**Lowest practical score** ^**b**^	68.00/68.00/72.00	80.00/84.00/87.00

a top three.

b last three.

#### 2. Scenario-based simulation receives higher professional evaluations.

**Fig 3:**
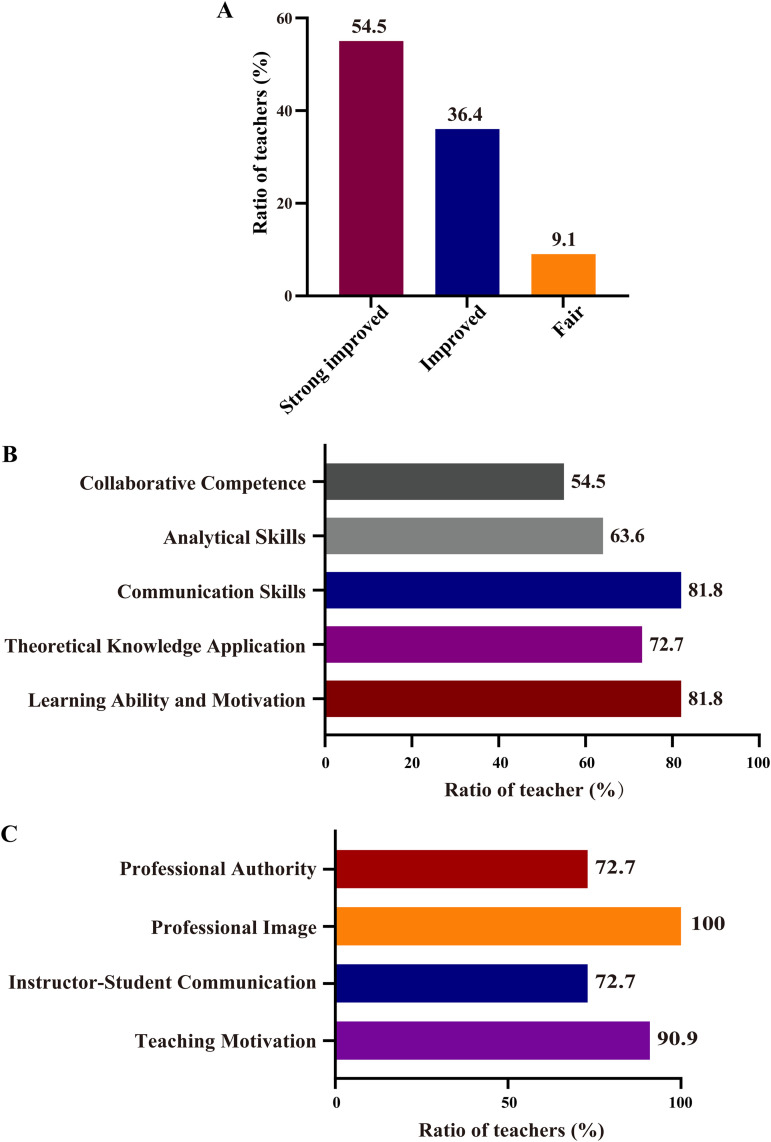
Professional teacher evaluations comparing scenario-based simulation teaching with traditional teaching. Student engagement and motivation (A), Professional competency development (B), Expertise utilization (C). n = 11.

Professional instructors (n = 11) assessed scenario-based simulation versus traditional teaching in terms of engagement, motivation, and instructional efficacy. Most instructors agreed that scenario-based simulation improved student engagement and motivation (Fig 3A) and was more effective in developing professional competencies (Fig 3B).

Key instructor-reported benefits included:

1) Learning Ability and Motivation: 81.8% noted significant improvements in student willingness and learning capacity, attributing this to realistic simulations fostering intrinsic motivation.
2) Theoretical Knowledge Application: 72.7% observed better practical application of theory, bridging classroom and real-world problem-solving.
3) Communication Skills: 81.8% reported more active group discussions, enhancing expressive and listening abilities.
4) Analytical Skills: 63.6% noted marked improvements in critical reasoning during complex simulations.
5) Collaborative Competence: 54.5% observed enhanced team efficiency during simulation activities.

All instructors agreed that scenario-based teaching optimized their expertise utilization (Fig 3C), with additional benefits including:

1) Teaching Motivation (90.9%): Innovative methods and positive feedback reinvigorated instructor enthusiasm.
2) Instructor-Student Communication (72.7%): Frequent simulation interactions during strengthened rapport.
3) Professional Image (100%): Successful implementation elevated perceived expertise and professional identity.
4) Professional Authority (72.7%): Active guidance during simulations reinforced their authoritative role.

#### 3. Student self-assessment favors scenario-based simulation.

Students self-assessed theoretical learning effectiveness (TLE), analytical/operational capabilities (AOC), and professional self-assurance (PSA) under both teaching methods.

**Fig 4:**
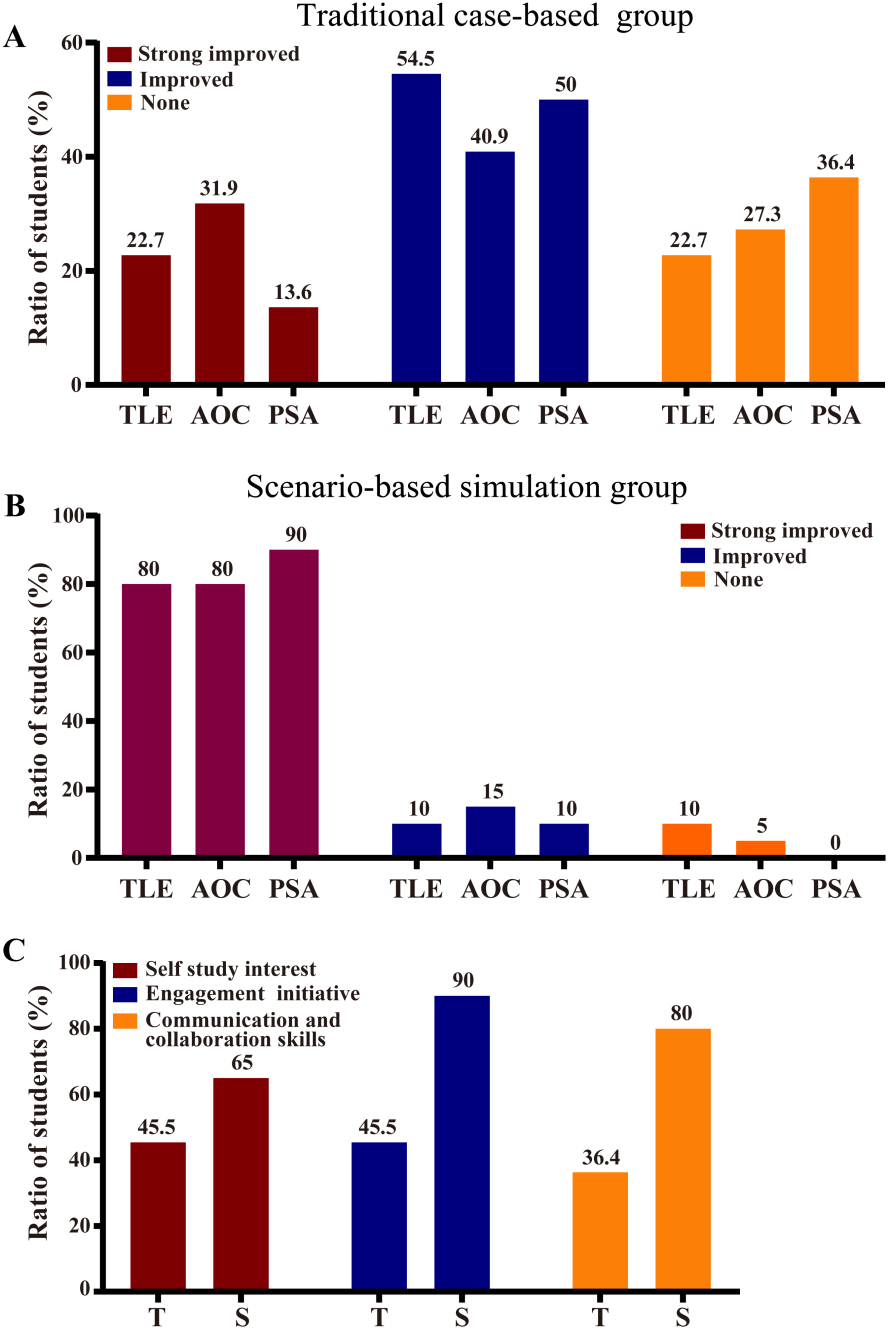
Student self-assessments of learning experiences. (A) Traditional group and (B) situational group evaluations of theoretical learning efficiency (TLE), analytical/operational competence (AOC), and professional self-assurance (PSA). (C) Comparative self-ratings of learning interest, engagement motivation, and collaborative communication skills across instructional modes. T: Traditional group (n = 22), S: Situational group (n = 20).

Traditional Teaching Group: Only 22.7%, 31.9%, and 13.6% of students strongly agreed that traditional teaching improved TLE, AOC, and PSA, respectively. Over 40% reported limited effectiveness, with 22.7%, 36.4%, and 27.3% noting no improvement in these areas (Fig 4A).

Scenario-Based Simulation Group: Most students endorsed the method for its optimized learning environment and structured curriculum (Fig 4B). Over 80% reported significant improvements in theoretical retention and conceptual familiarity, with only 10% perceiving negligible differences and 5% expressing negative attitudes (Fig 4B).

Classroom dynamism: The simulation group showed higher self-study interest (65% *vs.* 45.5%), engagement initiative (90% *vs.* 45.5%), and communication/collaboration skills (80% vs. 36.4%) compared to the traditional group. A minority (10%) resisted due to increased preparation demands and complex in-class tasks (Fig 4C).

## Discussion

### 1. Teaching challenges in forensic clinical medicine

Global forensic medicine education systems exhibit common deficiencies, including insufficient curricular focus, limited practical resources, and minimal exposure to real cases. These issues have become more pronounced with technological advancements and growing public legal awareness. In China, traditional lecture-based approaches in forensic clinical programs inadequately develop the professional competencies required for injury assessment, medical malpractice investigations, and sexual assault examination [12]. Student feedback consistently highlights gaps in critical skill training, particularly in examinee evaluation and diagnostic analysis, which hinder professional preparedness after graduation [13].

While educators have introduced reforms to improve workplace competence, most innovations remain restricted to case-based learning models [14,15], leaving the theory-practice gap unresolved. Effective pedagogical reform should encompass the full forensic clinical workflow-from case intake to expert report issuance-rather than isolated knowledge points or situational exercises. The persistent disconnect between theory and practice continues to impede competency development [15].

Scenario simulation teaching proves more effective than fragmented case analyses or technical drills by integrating theory, practical skills, and real-world contexts. This established medical education method enhances analytical abilities through problem-solving, operational proficiency through hands-on practice, and job readiness through realistic simulations [16].

### 2. Advantages of scenario-based simulation in forensic clinical practice

Scenario-based simulation, used in medical education since the 1950s, has demonstrated pedagogical benefits across disciplines [17,18]. Our implementation in forensic clinical curricula confirms its dual advantages for educators and learners. The approach improves engagement, legal awareness, theoretical mastery, and skill application, with benefits manifesting in three key areas.

**First,** **it increases learning motivation and participation.** Simulating real workflows-such as injury assessment and examinee interviews-boosted student engagement and self-directed learning. Eighty percent reported better knowledge retention and conceptual understanding compared to traditional methods, while fewer than 10% perceived negligible differences (Fig 3B). Role-playing exercises, including expert-simulated examinees, enhanced immersion, shifting students from passive recipients to active learners.

**Second**, it improves knowledge integration and skill development. Most students favored scenario-based designs, though 10% cited additional preparation demands. The task-driven approach bridges theory and practice, enabling students to apply medical and forensic knowledge in complex situations (e.g., uncooperative examinees, conflicting evidence), facilitating the transition from conceptual understanding to practical application. This aligns with Guo et al.’s findings on practice-enhanced learning [5] and correlates with superior performance in theoretical and operational assessments.

**Third**, it fosters comprehensive professional competencies. Scenario simulations systematically train both medical diagnostic reasoning and judicial evidence awareness. Immediate feedback mechanisms (e.g., instructor and peer evaluations) create a closed-loop learning cycle: “theory → simulation → feedback → internalization.” Students also refined clinical adaptability, critical thinking, and ethics when navigating challenges like communication barriers, while minimizing ethical risks inherent in real examinations.

Comparative evaluation confirms scenario simulation’s stronger impact on knowledge acquisition, retention, and skill application than traditional teaching. The high correlation between teaching effectiveness and final grades (*r* = 0.9606) supports this approach, resonating with Miller’s Pyramid framework [19]. Such integration is critical in forensic medicine, where practitioners must master both theory and practice.

Our findings align with established medical education research [20–22] while uniquely demonstrating scenario simulation’s dual benefits in forensic medicine: reinforcing knowledge-skill integration and cultivating judicial practice competencies. This provides empirical support for curricular reform.

### 3. Limitations

The study has several limitations. First, the small sample size (n = 42) from two student cohorts at Harbin Medical University necessitates broader participation and more diverse cases for robust evaluation. Second, the short tracking period for graduate performance requires extended longitudinal follow-up. Third, self-assessment data may reflect response bias.

Practical implementation barriers include high equipment and maintenance costs for simulation laboratories, compounded by forensic medicine’s multi-system examination requirements. Interdepartmental collaboration to develop shared resource centres could mitigate these challenges.

Additionally, a minority of students resistant to simulation-based learning exhibited passive or oppositional attitudes. Large-scale adoption should accommodate diverse preferences through optional teaching groups, allowing students to compare methodologies for optimal outcomes.

### 4. Future directions

Scenario-based simulation should transition from a supplementary tool to a core component of forensic clinical education. Future research should:

1) Conducting longitudinal studies on educational outcomes.
2) Developing standardized forensic competency assessments.
3) Optimize resource allocation strategies.
4) Establish adaptive curricula for diverse student needs.
5) Expand a tiered simulation case library, ranging from single-injury assessments to complex multifactorial cases.

## Conclusions

Scenario-based teaching using real cases significantly improved students’ knowledge acquisition, practical skills, and professional competencies in forensic clinical practice. By strengthening theory-practice integration, the method enhances problem-solving abilities for real-world forensic challenges. The results affirm its effectiveness in applied disciplines like forensic medicine, equipping students with the analytical and practical skills needed for their future roles.

## Supporting information

S1 Data Source data for Fig 2. (PDF)

S2 Data Source data for Fig 3. (PDF)

S3 Data Source data for Fig 4. (PDF)
