# Supplementary material for: Enhancing forensic clinical competence through scenario-based simulation: A comparative study of educational outcomes in Chinese medical students
Source: PLoS One. 2025 Nov 13;20(11):e0336273. doi: 10.1371/journal.pone.0336273 (PMC12614606; doi:10.1371/journal.pone.0336273)
Supplement: S1 Data — (PDF) [file pone.0336273.s001.pdf]

S1 Data. Source data for Figure 2.

Raw dataset used to generate the statistical charts and analyses presented in Figure 2A

Tradition	74	65	77	71	77	82	78	78	82	62	73	56	70	81	70	83	72	71	62	65	67	70
Simulation	64	93.75	95	96.25	94.25	80	79	86.75	91.25	90.25	95	95	90.25	85.25	91.25	86.5	80.25	99	89.25	84.25		

Raw dataset used to generate the statistical charts and analyses presented in Figure 2B

Tradition	86	72	90	81	90	98	92	92	78	68	84	59	80	86	80	99	83	81	68	72	75	80
Simulation	80	96	97	98	96	88	87	92	95	94	97	97	94	90	92	92	84	98	90	88		

Raw dataset used to generate the statistical charts and analyses presented in Figure 2C

Tradition	26.66667	16.66667	30	20	26.66667	31.66667	26.66667	28.33333	31.66667	18.33333	21.66667	20	25	33.33333	21.66667	31.66667	26.66667	26.66667	20	21.66667	18.33333	21.66667
Simulation	25	48.75	49	48.25	49.25	42	38	44.75	44.25	42.25	49	48	45.25	39.25	49.25	39.5	38.25	50	44.25	39.25		

Raw dataset used to generate the statistical charts and analyses presented in Figure 2D

Tradition	41.42857	39.28571	42.14286	42.14286	43.57143	45	44.28571	43.57143	45	36.42857	42.85714	31.42857	39.28571	43.57143	40.71429	45.71429	40	39.28571	35.71429	37.14286	40	40.71429
Simulation	39	45	46	48	45	38	41	42	47	48	46	47	45	46	42	47	42	49	45	45		

Raw dataset used to generate the statistical charts and analyses presented in Figure 2E

Tradition	74	93.75	95	96.25	94.25	80	79	86.75	91.25	90.25	95	95	90.25	85.25	91.25	86.5	80.25	99	89.25	84.25
Simulation	80	96	97	98	96	88	87	92	95	94	97	97	94	90	92	92	84	98	90	88

Raw dataset used to generate the statistical charts and analyses presented in Figure 2F

Tradition	81	76	83	79	83	86	84	84	86	74	81	70	79	85	79	87	80	79	74	76	77	79
Simulation	86	72	90	81	90	98	92	92	78	68	84	59	80	86	80	99	83	81	68	72	75	80
