# Supplementary material for: Enhancing forensic clinical competence through scenario-based simulation: A comparative study of educational outcomes in Chinese medical students
Source: PLoS One. 2025 Nov 13;20(11):e0336273. doi: 10.1371/journal.pone.0336273 (PMC12614606; doi:10.1371/journal.pone.0336273)
Supplement: S2 Data — (PDF) [file pone.0336273.s002.pdf]

S2 Data. Source data for Figure 3.

Raw dataset used to generate the statistical charts and analyses presented in Figure 3A

Strong improved	improved	Fair
55	36	9

Raw dataset used to generate the statistical charts and analyses presented in Figure 3B

Learning Ability and Motivation	Theoretical Knowledge Application	Communication Skills	Analytical Skills	Collaborative Competence
82	73	82	64	55

Raw dataset used to generate the statistical charts and analyses presented in Figure 3C

Teaching Motivation	Instructor-Student Communication	Professional Image	Professional Authority
91	73	100	73
