# Supplementary material for: Enhancing forensic clinical competence through scenario-based simulation: A comparative study of educational outcomes in Chinese medical students
Source: PLoS One. 2025 Nov 13;20(11):e0336273. doi: 10.1371/journal.pone.0336273 (PMC12614606; doi:10.1371/journal.pone.0336273)
Supplement: S3 Data — (PDF) [file pone.0336273.s003.pdf]

S3 Data. Source data for Figure 4.

Raw dataset used to generate the statistical charts and analyses presented in Figure 4A

	Strong improved	Improved	None
TLE	22.7272727	54.5454545	22.7272727
AOC	31.8181818	40.9090909	27.2727273
PSA	13.6363636	50	36.3636364

Raw dataset used to generate the statistical charts and analyses presented in Figure 4B

	Strong improved	Improved	None
TLE	80	10	10
AOC	80	15	5
PSA	90	10	0

Raw dataset used to generate the statistical charts and analyses presented in Figure 4C

	Self study interest	Engagement initiative	Communication and collaboration skills
Tradition	45.4545455	45.4545455	36.3636364
